# Relapse of Euglycemic Diabetic Ketoacidosis With Prolonged Glucosuria and Ketonuria Associated With Pre-operative Sodium-Glucose Co-transporters 2 (SGLT-2) Inhibitor Use

**DOI:** 10.7759/cureus.90403

**Published:** 2025-08-18

**Authors:** Basel Alheijani, Ahmad T Alswaidan, Osama T Al Swaidan, Ahmad B Alheijani

**Affiliations:** 1 Internal Medicine, King Abdulaziz Medical City, Riyadh, SAU; 2 Medical College: Surgery, Alfaisal University College of Medicine, Riyadh, SAU

**Keywords:** euglycemic diabetic ketoacidosis, glucosuria, persistent, relapsing, sodium-glucose cotransporter 2 inhibitors

## Abstract

Sodium-glucose co-transporters 2 (SGLT2) inhibitors are an important class of medications that have become increasingly used not only in the management of type 2 diabetes but also for cardioprotection and slowing the progression of renal dysfunction. However, clinicians should be aware of the risk of euglycemic diabetic ketoacidosis associated with their use, particularly in cases of severe illness, volume depletion, decreased carbohydrate intake, or recent surgery.

We present a case of a 68-year-old male, whose type 2 diabetes mellitus was controlled with dapagliflozin 10 mg daily, and was electively admitted for left tibial fracture surgery. He continued to take dapagliflozin until one day prior to the scheduled surgery date and was discharged home the day after the procedure. Twelve hours after discharge, he presented again to the emergency room with severe euglycemic diabetic ketoacidosis (EuDKA) and acute kidney injury (AKI), which required intensive care admission and initiation of the diabetic ketoacidosis (DKA) protocol. Despite successful treatment of EuDKA and AKI, and subsequent transfer to the medical floor, his urinalysis showed persistent ketonuria and glucosuria, even though blood sugar readings remained below the renal threshold of glucosuria. On the medical floor, euglycemic diabetic ketoacidosis recurred eight days after the last dose of dapagliflozin. The second EuDKA episode resolved following another 24 hours of treatment with the DKA protocol. The patient’s hospital stay was complicated by partial deep thrombosis of the distal superficial femoral vein (DVT), which was treated with apixaban. He was discharged in stable condition while his urinalysis was still showing persistent glucosuria and ketonuria, with near-normal serum blood glucose levels after 11 days of dapagliflozin cessation.

## Introduction

Diabetic ketoacidosis (DKA) is a life-threatening complication of diabetes mellitus. It usually occurs when an absolute insulin deficiency leads to decreased glucose utilization, enhanced lipolysis, and increased delivery of free fatty acids (FFAs) to the liver, where they are converted into ketone bodies [1].

While DKA in both type 1 and type 2 diabetes presents with marked hyperglycemia, typically between 350 and 800 mg/Dl, euglycemic DKA (EuDKA) was originally defined as DKA occurring with a plasma glucose level of less than 200 mg/ dl, found mainly in T1D. The primary cause was reduced bioavailability of carbohydrates, usually occurring alongside a reduced insulin dose [2].

EuDKA associated with sodium-glucose co-transporter 2 (SGLT2) inhibitor use has a different and unique mechanism. SGLT2 inhibitors, such as dapagliflozin, reduce the reabsorption of glucose at the proximal renal tubule, increasing glucosuria, which leads to reduced plasma glucose. Reduced insulin production from pancreatic β (beta) cells and increased volume depletion risk, in addition to SGLT2 inhibitors, may increase plasma glucagon levels via a direct effect on pancreatic alpha-cells. This leads to enhanced hepatic ketogenesis and increased risk of ketoacidosis, while maintaining near-normal glucose levels [3,4].

Reported rates of euglycemic diabetic ketoacidosis during clinical trials have been rare: 0.2 to 0.6 per 1,000 patient-years for empagliflozin 10 mg and 25 mg, respectively, and 0.76 and 0.24 per 1,000 patient-years for canagliflozin 100 mg and 300 mg, respectively [1].

To minimize the risk of developing EuDKA, the U.S Food and Drug Administration (FDA) and American College of Cardiology (ACC) recommend withholding SGLT2 inhibitors in case of severe illness and for three to four days prior to surgery. It has not been recognized that the effect of SGLT2 inhibitors persists beyond the five half-lives of drug discontinuation [2-5].

The objective of this report is to describe a case of relapsing EuDKA, along with prolonged glucosuria and ketonuria, associated with the use of an SGLT2 inhibitor prior to surgery, and to discuss potential factors contributing to the prolonged effects of this drug class beyond the expected duration of five half-lives after discontinuation.

## Case presentation

**Figure 1:**
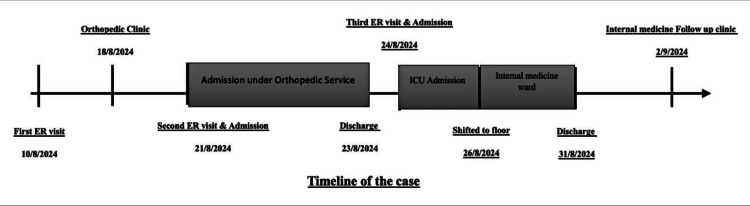
Timeline of the case

This was a 68-year-old Saudi male with a history of type 2 diabetes mellitus, well-controlled on dapagliflozin 10 mg once daily, with no other home medications. He had been in good health until August 10, 2024, when he presented to the emergency room (ER) complaining of left leg pain following a fall down the stairs while in a flexible position, as shown in Figure 1.

**Figure 2:**
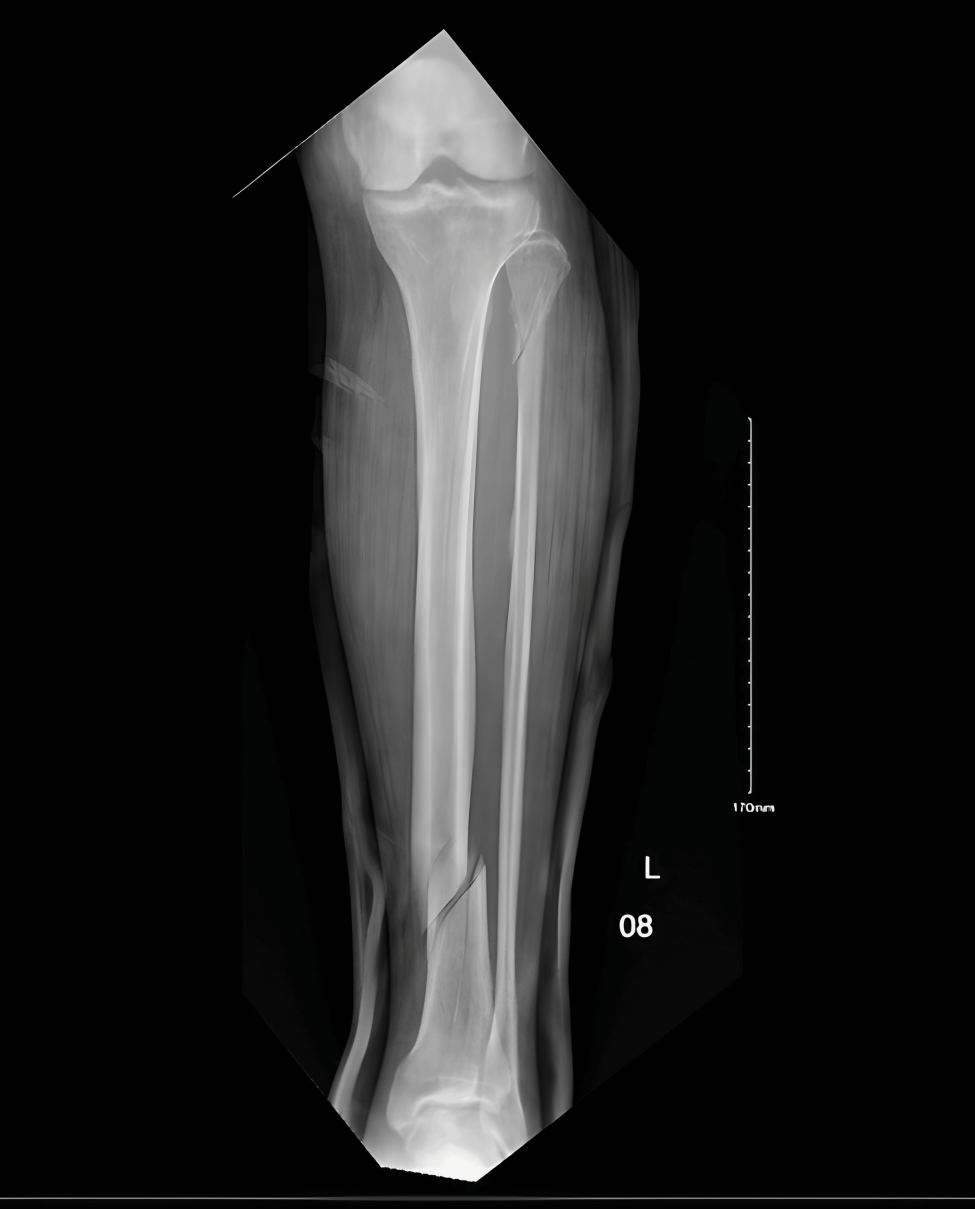
Upon the visit to the Emergency Room on 10/8/2024 (before the backslabs)

His tibial and fibular X-ray revealed distal tibial and proximal fibular fractures. The orthopedic team on call conducted an evaluation and arranged for his admission for surgical management. He declined the surgical intervention, however, and asked to apply backslabs along with pain management. The patient was discharged DAMA (Discharge Against Medical Advice), and close clinic follow-up was given as shown in Figure 2.

**Figure 3:**
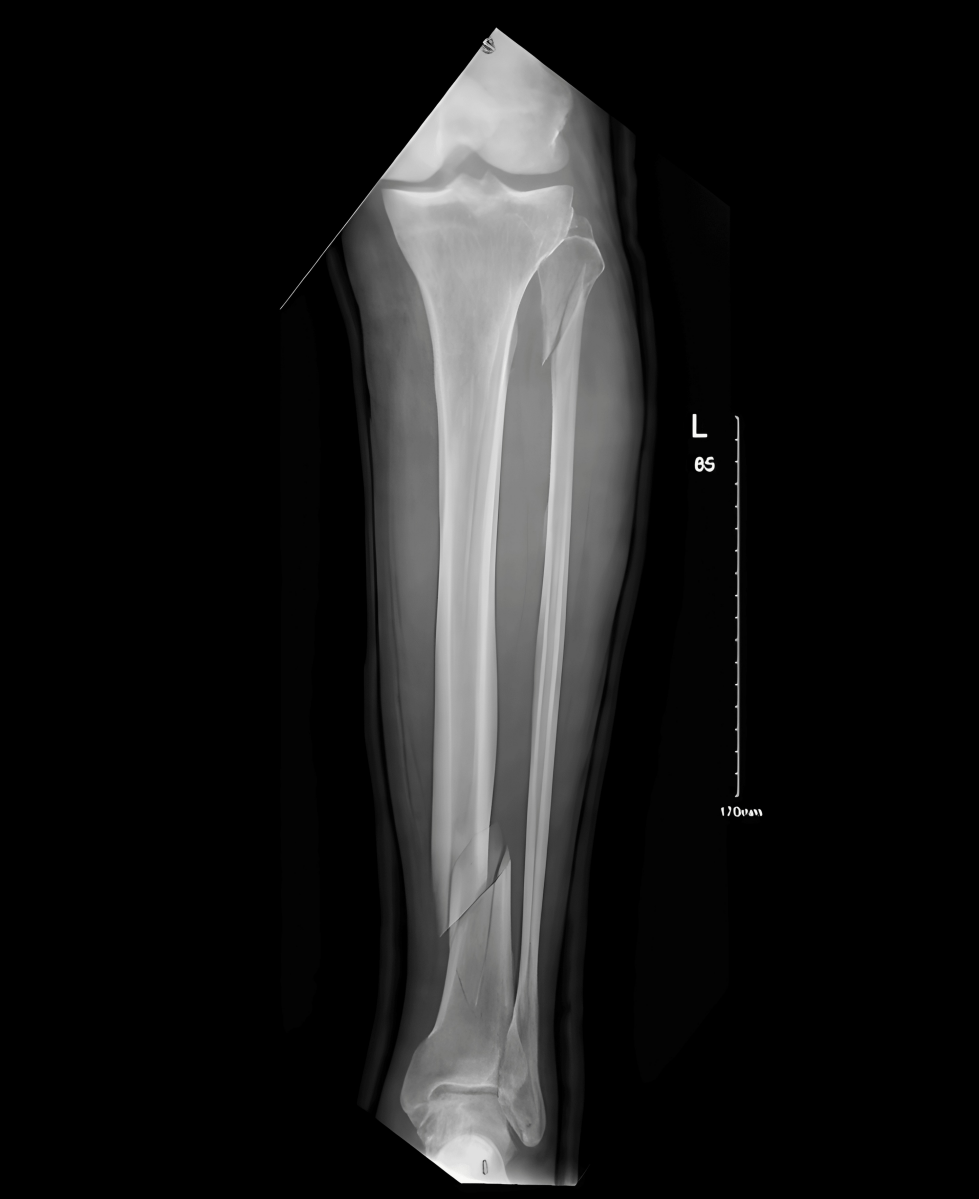
In the orthopedic clinic on 18/8/2024 (after removal of the backslabs)

After eight days, he came back to the orthopedic clinic, where it was found that the fractures had failed to reduce with backslabs as shown in Figure 3.

**Figure 4:**
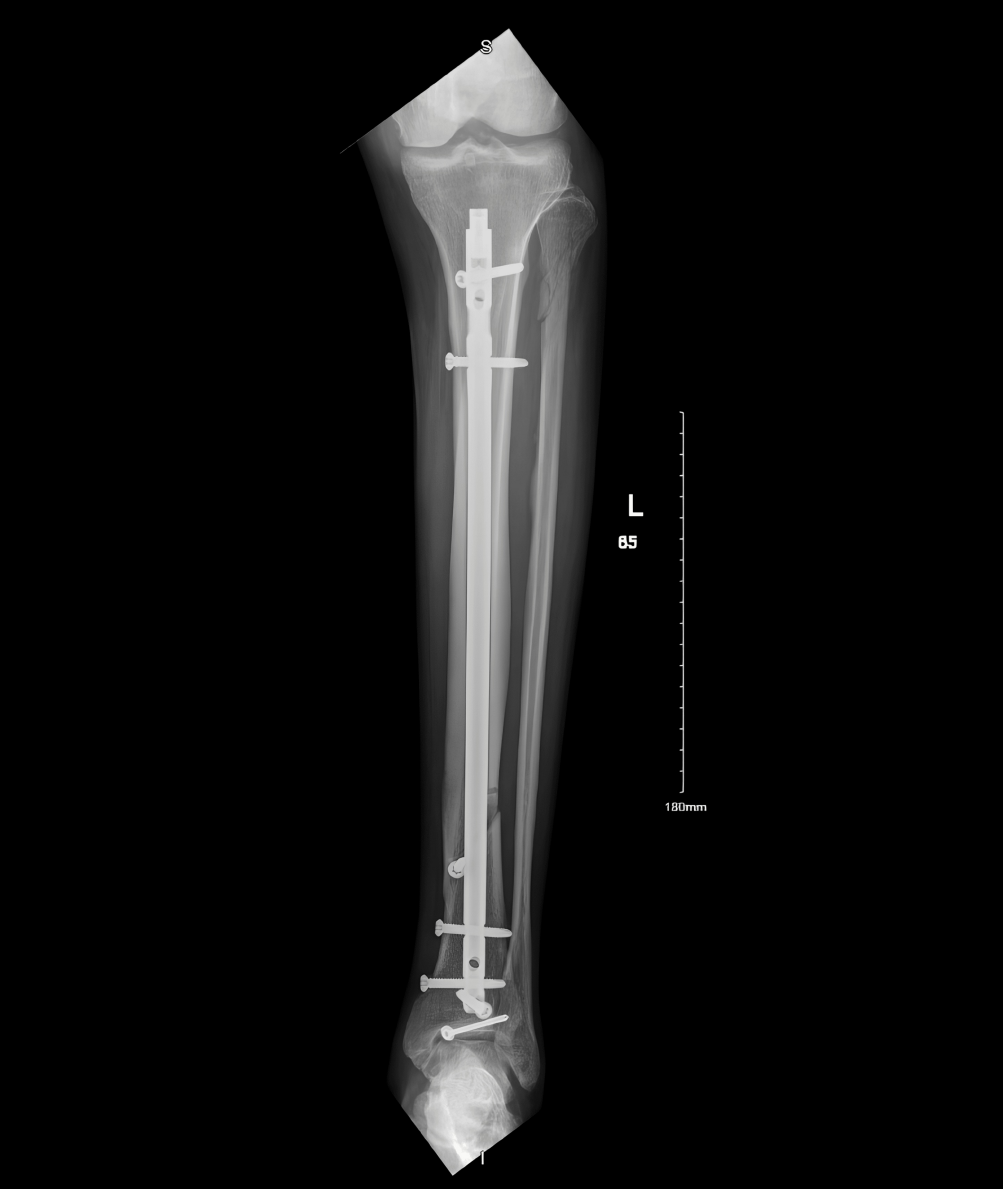
After fracture fixation on 8/10/2024

The patient agreed to proceed with surgical intervention. Three days after that, on 21/8/2024, he was admitted electively under the orthopedic service for open reduction and internal fixation (ORIF). Surgery under general anesthesia was scheduled for the next day (22/8/2024) as shown in Figure 4. The patient continued to receive his usual dose of SGLT2 inhibitors (dapagliflozin 10 mg daily) until one day prior to hospital admission; it was discontinued after that. His blood sugar readings ranged from 10 to 13 mmol/L (180-230 mg/dL) on the day of admission, and glycated hemoglobin (HbA1c) was 7.1% (normal range: 4-5.6%).

As preparation for surgery, the patient was commenced on the routine surgical protocol, which consists of frequent blood glucose monitoring, IV insulin infusion, and IV fluid dextrose 5% in water or normal saline, accordingly. In addition, he was started on enoxaparin 30 mg BID for deep vein thrombosis (DVT) prophylaxis and an antibiotic (clindamycin), as he was allergic to penicillin. He underwent successful left tibial surgery and nail implant with no immediate complications. The next day, he was vitally and clinically stable, with a blood sugar reading ranging from 8-10 mmol/L (144-180 mg/dl) during and after surgery. The patient was discharged one day after surgery on 23/8/2024. His discharge medications included: apixaban prophylaxis dose (2.5 mg) twice daily, pain killers (tramadol, acetaminophen), and to resume the SGLT2 inhibitor (dapagliflozin) home dose. Despite being clinically stable with an intact surgical site, his labs the day of discharge showed some evidence of metabolic acidosis (bicarbonate was 12 mmol/L and anion gap was 24 mEq/L, with no documented blood gas). Blood sugar was 10 mmol/l (180 mg/dl), and there was no evidence of sepsis or infection. 

After around 12 hours of discharge, on 24/8/2024, the patient was brought back to the ER because of persistent vomiting, tachypnea, and agitation. Upon initial examination, the patient looked distressed, tachypneic (30 breaths/minute), tachycardic (130 beats/minute), hypertensive, with a blood pressure reading of 171/91 mmHg. His physical examination was unremarkable, and his surgical site showed no pus, erythema, crepitus, or any signs of infection. His initial venous blood gas (VBG) results indicated severe high anion gap metabolic acidosis (pH 7.04, CO2 22 mmHg, HCO3 5.9 mEq/L, Na 139 mEq/L, Cl 105mEq/L). Blood glucose stood at 12 mmol/L (216 mg/dl). Creatinine level upon admission was 102, but upon discharge, it was 50. Urinalysis revealed ketones >150 mg/dL and glucosuria >1,000 mg/dL with no evidence of urinary tract infection (UTI) as shown in Table 1.

**Table 1:** Summary of the labs and medications during hospitalization and after discharge ICU: Intensive Care Unit; IM: Internal Medicine; pH: Potential of Hydrogen; CO2: Carbon Dioxide; HCO3: Bicarbonate; DM: Diabetes Mellitus; DKA: Diabetic Ketoacidosis *Calculated AG: Calculated anion gap = 2*Na - (HCO3+ Cl) **Normal value of anion gap is based on albumin level. To calculate the lower normal range, we should multiply the Albumin level (g/dL) by 2. Furthermore, we can multiply the albumin level (g/dl) by 3 for the upper normal range.

Location/Labs		During Admission								After Discharge
Parameter	Normal range	ICU Admission		IM Floor						IM Clinic
Unit		24/8	25/8	26/8	27/8	28/8	29/8	30/8	31/8	1/9
Venous Blood Gas										
											pH	7.35-7.45	7.04	7.32	7.35	7.37	7.31	7.33	7.38	7.46	Not Available
HCO3	22-26 mEq/L	5.9 mEq/L	16.5 mEq/L	18.2 mEq/L	14.5 mEq/L	13.1 mEq/L	16.9 mEq/L	17.7 mEq/L	22 mEq/L	Not Available
CO2	35-45 mmHg	22 mmHg	32 mmHg	33 mmHg	25 mmHg	26 mmHg	32 mmHg	30 mmHg	31 mmHg	Not Available
Electrolytes and Lactic Acid										
											Na+	135-145 mEq/L	139 mEq/L	145 mEq/L	144 mEq/L	141 mEq/L	137 mEq/L	139 mEq/L	142 mEq/L	140 mEq/L	137 mEq/L
K+	3.5-5.2 mEq/L	5.01 mEq/L	3.7 mEq/L	3.5 mEq/L	3.7 mEq/L	3.9 mEq/L	4.1 mEq/L	4 mEq/L	4.1 mEq/L	4.2 mEq/L
Cl-	96-106 mEq/L	108 mEq/L	121 mEq/L	115 mEq/L	112 mEq/L	114 mEq/L	116 mEq/L	114 mEq/L	111 mEq/L	106 mEq/L
Calculated AG*	Variable** mEq/L	Elevated	Normal	Elevated	Elevated	Elevated	Normal	Elevated	Normal	Not Available
		25 mEq/L	7.5 mEq/L	10.8 mEq/L	14.5 mEq/L	10 mEq/L	6.1 mEq/L	10.7 mEq/L	7 mEq/L	
Lactic Acid	0.5-2.2 mmol/L	5.2 mmol/L	0.73 mmol/L	Not Available	0.89 mmol/L	0.77 mmol/L	Not Available			
Renal Profile										
											Creatinine	60-110 μmol/L	149 μmol/L	76 μmol/L	65 μmol/L	71 μmol/L	53 μmol/L	56 μmol/L	50 μmol/L	51 μmol/L	50 μmol/L
Albumin	35-55 g/L	41 g/L	27 g/L	27 g/L	29 g/L	29 g/L	27 g/L	26 g/L	26 g/L	Not Available
Bicarbonate	22-26 mEq/L	>5 mEq/L	17 mEq/L	15 mEq/L	11 mEq/L	13 mEq/L	17 mEq/L	17 mEq/L	19 mEq/L	19 mEq/L
Urine Analysis										
											Urine Ketones	0-20 mg/dL	150 mg/dL	Not Available			150 mg/dL	80 mg/dL	100 mg/dL	60 mg/dL	60 mg/dL
Urine Glucose	0-15 mg/dL	>1000 mg/dL	1000 mg/dL	>1000 mg/dl	>1000 mg/dl	>1000 mg/dl	>1000 mg/dl									
Current DM medications										
											DM Medications (During hospitalization and upon discharge)		DKA Protocol	Subcutaneous Aspart (5 Units TID) + Glargine (10 Units)	Metformin (500mg TID) + Sitagliptin (100mg OD) + intermitted aspart sliding scale	Subcutanous Aspart (4 units TID) + Glargine (8 units)	DKA Protocol + Glargine (8 Units)		Subcutanous Aspart (4 units TID) + Glargine (8 units)	Glargine (8 Units) + Metformin (500 mg TID)	

His renal profile also showed elevated creatinine (149 μmol/L), elevated potassium (5.3 mEq/L), significantly low bicarbonate (<5 mEq/L), and elevated lactic acid (3.82 mmol/L) despite his claim that he did not receive any dapagliflozin after discharge from the hospital. The patient was admitted as a case of severe euglycemic DKA due to SGLT2I use. He was started on DKA protocol, including intravenous insulin drip, potassium replacement, and adequate fluid hydration, in addition to serial glucose and venous blood gases monitoring. A septic workup was sent, and the patient was transferred to the ICU for continuity of care.

During his ICU stay, he received vancomycin and ciprofloxacin empirically because of his significant leukocytes (WBC 24.5*10^9/L) (normal range: 4.5-11* 10^9/L) and elevated inflammation markers (PCT 0.56 μg/L (normal range: less than 0.1 μg/L), ESR 91 mm/hr (normal range: less than 20 mm/hr, and CRP 273.50 mg/L (normal range: less than 1 mg/L)). During the second day of his ICU admission, and after approximately 24 hours of the DKA protocol, he was able to tolerate oral intake with no vomiting. His anion gap had closed, his blood sugar reading was 9 mmol/dl (162 mg/dl), and his kidney function had improved, which led to normalization of his creatinine levels. The DKA protocol was stopped and switched to subcutaneous insulin aspart 5 units before each meal and insulin glargine 10 units at bedtime.

All septic workup came back negative, with no evidence of infection; however, his urinalysis showed persistent glucosuria and ketonuria since admission, even after DKA resolution. Antibiotics were discontinued, and the patient was transferred from the ICU to the medical floor on 25/8/2024, in stable condition. After one day, the patient underwent venous Doppler ultrasound, which revealed partial thrombosis of the distal superficial femoral vein (DVT) after he complained of right leg pain and swelling. Consequently, he was commenced on apixaban -- full anticoagulation dose.

The endocrine team was consulted for further blood sugar management after DKA had resolved with persistent glucosuria and ketonuria. Initially, they recommended replacing basal and prandial insulin with oral diabetic medications, including metformin 500 mg three times a day, sitagliptin 100 mg once daily, and intermittent doses on a sliding scale every four hours. However, the patient was unable to tolerate oral hypoglycemics due to dyspepsia; therefore, basal and prandial insulin were reinitiated, along with esomeprazole. Sitagliptin was added after dyspepsia resolved.

On August 28, 2024, after almost 48 hours of EuDKA resolution and 8 days of the last dapagliflozin dose, and while the patient was already on glargine, aspart, and sitagliptin, his daily labs showed a low PH (7.31), a low bicarbonate (11 mmol/L), and an elevated anion gap of 21. Glucose level was only 7.7 mmol/L (138 mg/dl), and his urinalysis showed significant ketonuria (150 mg/dl) and glucosuria (1000mg/dl) yet again. Creatinine and lactic acid levels were normal.

As this lab was suggestive of relapsed euglycemic DKA, the patient was started again on the DKA protocol, which required almost another 24 hours to resolve. On August 30, 2024, the labs revealed a normal PH of 7.41 and serum bicarbonate of 18 mmol/L, with a closed anion gap and blood sugar of 7 mmol/L. The patient was vitally and clinically stable. Despite his assuring labs, repeated urine analysis showed persistent and significant ketonuria and glucosuria, which was suggestive of persistent SGLT2I effect even after 10 days of dapagliflozin discontinuation. His kidney function was normal, there was no clinical or laboratory evidence of infection, and he was started again on basal and pre-prandial insulin. The nephrology team was consulted for any possibility of renal causes of persistent ketonuria and glucosuria, such as renal tubular acidosis, and all were excluded.

After resolving the second episode of EuDKA, the patient was kept for another 24 hours for evaluation. He was clinically stable and tolerating oral intake. After discussion between the treating teams -- including internal medicine, endocrinology, and orthopedics -- a decision was made to discharge the patient home with close follow-up in the internal medicine clinic two days later. A repeat urinalysis was planned to assess persistent glucosuria and ketonuria, which had been present up to the day of discharge on August 31, 2024.

 Discharge medications included metformin 500 mg three times a day, glargine 8 units once daily at bedtime for diabetes, and apixaban 5 mg twice a day for three months for provoked DVT. The patient did not attend his internal medicine clinic appointment; however, a urinalysis performed on 1/9/2024, one day after discharge from the hospital, was still showing a urine ketone level of 60 mg/dL and urine glucose exceeding 1000mg/dL, despite consistently normal blood glucose levels below the renal threshold. His son, who attended the appointment in his place, reported that the patient was clinically stable and doing well at home following discharge.

## Discussion

Our case of euglycemic DKA occurred one day after dapagliflozin cessation and orthopedic surgery, with recurrence of DKA after eight days of SGLT2 inhibitor elimination. His recurrent DKA was accompanied by persistent glucosuria and ketonuria for more than 10 days after the latest dose of dapagliflozin, highlighting a potentially prolonged pharmacological effect of SGLT2 inhibitors. The patient had elevated creatinine on admission, which improved on IV fluids, and the hospital stay was complicated by lower limb DVT with no evidence of infection.

Current guidelines recommend against the routine use of SGLT2 inhibitors during hospitalization and advise avoiding their use in cases of severe illness and prolonged fasting [2]. In addition, the FDA recommends the discontinuation of SGLT2 inhibitors at least three to four days prior to surgery. However, these guidelines do not acknowledge the potential effects of SGLT2 inhibitors to persist for more than five half-lives following elimination [2]. In most cases, SGLT2 inhibitor-associated euglycemic DKA and glucosuria usually resolve within a few days of treatment [6], with urinary glucose levels returning to baseline approximately three days after drug discontinuation [7]. However, there have been several reports where euglycemic DKA required insulin therapy for one to two weeks following SGLTI cessation, as shown in Table 2 [3,8-13].

**Table 2:** Summary of some published cases with prolonged or recurrent euDKA DKA: Diabetic Ketoacidosis; euDKA: Euglycemic DKA

Reference number	Author Name	Age/Gender	SGLT2 Inhibitors	Duration of Metabolic Recovery	Duration of Glucosuria	Recurrence of Euglycemic DKA
							[9]	Pujara et al.	50 years old	Dapagliflozin	9 days	9 days	Yes
Female													
[10]	Fukuda et al.	71-year-old	Canagliflozin	6 days	12 days	No
		Female				
[11]	Rafey et al.	59-year-old	Empagliflozin	92 hours	Not Documented	Yes
		Female				
[12]	Solan et al.	63-year-old	Canagliflozin	12 days	Not Documented	Yes
		Male				
[13]	Yehya et al.	57-year-old	Empagliflozin	9 days	14 days	Yes

The etiology of the disconnect between plasma SGLT2I concentrations and their pharmacodynamic response has not been fully understood, yet some factors may contribute to the prolonged effects of glucosuria and ketosis beyond the drug clearance period of five half-lives, like a decrease in renal function, which leads to a longer drug half-life, higher drug affinity to lipids, and an enhanced ability to dissolve in them (lipophilicity), especially in older people with increased adiposity, which could expand the distribution volume of the drug and increase the drug elimination half-life [3,14]. Another contributing factor is the slow off-rate of SGLT2 inhibitors. These agents can stay bound to the SGLT2 transporter in the kidney for a prolonged period and do not detach even after plasma drug concentrations decrease, potentially resulting in sustained glucosuria and increased risk of complications such as euDKA [15].

In our patient, the main triggering factor of the development of euDKA after surgery appears to be non-compliance with the guidelines and recommendations to hold SGLT2 inhibitors for a few days (three to four) prior to surgery. Other possible factors include surgical stress, low carbohydrate intake, and inadequate insulin therapy in the perioperative period. In addition, the patient had acute kidney injury on presentation to the ER, which may have partially reduced the excretion of the drug and prolonged its half-life. However, the relapse of euDKA and persistent ketonuria and glucosuria for over 10 days of elimination suggests a persistent dapagliflozin effect beyond the expected resolution period of two to three days, considering the estimated dapagliflozin half-life of 12.9 h, which raises the need for high clinical suspicion of such a rare complication of SGLT2 inhibitor use.

## Conclusions

This case highlights an important issue in patients treated with SGLT2 inhibitors. A careful preoperative evaluation, compliance with the guidelines of holding SGLT2I a few days before surgery, timely initiation of basal insulin in acutely ill patients and the perioperative period, and assurance of adequate carbohydrate intake are all essential steps to avoid the underrecognized entity of euDKA in the absence of marked hyperglycemia. Furthermore, this case highlights the rare possibility of the prolonged effects of SGLT2 inhibitors lasting more than a week after discontinuation, as well as the risk of relapsing euDKA, particularly in the context of surgery, low carbohydrate intake, acute illness, and impaired kidney function.

Inpatient physicians should be aware of the potential for prolonged glucosuria associated with the use of this important and widely used class of medications. This requires close inpatient monitoring, adequate insulin therapy and carbohydrate intake, and timely management of acute illnesses. Further studies are needed for a deeper understanding of the exact mechanism of prolonged glucosuria and ketosis associated with SGLT2I use, given the worldwide expansion of its indication.
